# Editorial: New roles of neutrophils and granulocytic MDSC in autoimmune diseases, inflammation, and anti-microbial immunity

**DOI:** 10.3389/fimmu.2022.974062

**Published:** 2022-07-29

**Authors:** Dragana Odobasic, Clare Louise Hawkins, Marko Radic

**Affiliations:** ^1^Centre for Inflammatory Diseases, Department of Medicine, Monash Medical Centre, Monash University, Clayton, VIC, Australia; ^2^Department of Biomedical Sciences, University of Copenhagen, Copenhagen, Denmark; ^3^Department of Microbiology, Immunology and Biochemistry, University of Tennessee Health Science Center, Memphis, TN, United States

**Keywords:** neutrophils (PMNs), MDSC (myeloid-derived suppressor cells), infection, inflammation, autoimmunity

Neutrophils are known as the “first responders” that arrive at sites of infection and inflammation. There, they rapidly respond and attack microbes. However, recent discoveries reveal that neutrophils make a more multilayered contribution to the immune response (1). Neutrophils use an array of molecules to interact with other immune and non-immune cells. Consequently, neutrophils are important in shaping both innate and adaptive immune responses and are critical players in the protective immunity against pathogens as much as in the development and progression of various inflammatory and autoimmune diseases (2).

We now know that neutrophils can have both pro and anti-inflammatory functions, which are highly dependent on the timing, the context, and the type of signals they receive. They can promote inflammation through the release of microvesicles and various granule enzymes such as proteases and myeloperoxidase (MPO), as well as reactive oxidants, neutrophil extracellular traps (NETs), cytokines, and chemokines (3). The release of neutrophil enzymes and other bioactive macromolecules may introduce post-translational modifications into surrounding host tissues, in the process providing neo-antigens and contributing to tissue damage. Neutrophils also provide a rich source of autoantigens such as proteinase-3 and MPO, and autoimmunity to these causes severe inflammation of small blood vessels (4).

In contrast, neutrophils also have a critical role in the resolution of inflammation, often through their interactions with macrophages, and subsequent tissue repair. Notably, neutrophils can either positively or negatively regulate the development of adaptive immunity in secondary lymphoid organs through their effects on dendritic cells, T cells, and B cells (5). In some instances, it has become evident that neutrophils can even themselves act as antigen-presenting cells to activate T cells. Additionally, research in the last decade has uncovered that neutrophils, acting as immunosuppressive granulocytic myeloid-derived suppressor cells (MDSC), can promote cancer growth, whereas, in autoimmune diseases, neutrophils may have a protective role (6).

This Research Topic presents original research and reviews the rich contribution of neutrophils to immunity. We focus on the diverse roles neutrophils and granulocytic MDSC play as regulators of the immune system. The contributions in this Special Topic examine the implications of neutrophils in various aspects of inflammation, immunity against pathogens, and in cancer.

In this collection of articles, Haist et al. describe experiments that examine the effect of Ly6-G-targeted knock-down of β2 integrins on the severity of invasive pulmonary aspergillosis. The deficiency of neutrophil adhesion receptors leads to more extensive disease, less NET release, reduced cytokine and reactive oxygen production, and an increase in apoptosis compared to control animals.

The laboratory of Mihaila et al. examine the functional dichotomy between N1 and N2 neutrophils and draw attention to the contribution of the S100A9 alarmin to elevated N1 proinflammatory activity, chemotaxis and oxidative burst. The authors suggest that S100A9 inhibition may reduce inflammation.

Deerhake et al. use single cell transcriptomics to dissect aspects of pathway selection in neutrophils that respond to Cryptococcus by enhanced oxidation versus an alternative subset that delays apoptosis and increases cytokine secretion which activates dendritic cells and alveolar macrophages.

Piatek et al. pursue the puzzle of the reduced activation potential of neutrophils in HIV-infected individuals and determine that the levels and chromatin distribution of tri-methylated lysine 4 in histone H3 are linked to reduced anti-microbial functions and greater abundance of this chromatin mark along genes regulated by NF-kB.

Valadez-Cosmes et al. examined low-density neutrophils from non-small cell lung cancer (NSCLC) patients by using an unbiased flow cytometry screen of over 300 potential cell surface markers, and pinpoint CD36, CD41 and CD61 as reliable indicators of this disease-associated neutrophil population.

Mollenhauer et al. observed the enhanced participation of granulocytes in ischemia reperfusion injury and the remodeling of heart muscle in six-transmembrane protein of prostate 2 (Stamp2) deficient mice, which exhibited increased reactive oxygen production and greater MPO release, leading to impaired heart function post-injury.

An underappreciated anti-bacterial mechanism, the production of chlorinated lipids by neutrophil MPO, was examined by Amunugama et al. and evaluated with regard to bacterial killing.

Finally, review articles highlighted potential reasons for the inadequacy of neutrophils that attack but not eliminate pathogenic mycobacteria (Parker et al.), and profiled the emerging role of MDSC in promoting severe COVID-19 by exacerbating the pro-inflammatory cytokine milieu and reducing the efficacy of T lymphocytes (Rowlands et al.). The rich diversity of topics discussed in this Research Topic are an indication of the many emerging roles of neutrophils in immunity.

## Author contributions

The authors contributed equally and in mutual agreement to the reviews of the submitted manuscripts and the writing of the editorial.

## Conflict of interest

The authors declare that the research was conducted in the absence of any commercial or financial relationships that could be construed as a potential conflict of interest.

## Publisher’s note

All claims expressed in this article are solely those of the authors and do not necessarily represent those of their affiliated organizations, or those of the publisher, the editors and the reviewers. Any product that may be evaluated in this article, or claim that may be made by its manufacturer, is not guaranteed or endorsed by the publisher.
